# The Role of Language in Expressing Agentivity in Caused Motion Events: A Cross-Linguistic Investigation

**DOI:** 10.3389/fpsyg.2022.878277

**Published:** 2022-06-16

**Authors:** Hae In Park

**Affiliations:** Department of Educational Theory and Practice, University at Albany, State University of New York, Albany, NY, United States

**Keywords:** caused motion events, motion event construal, agentivity, agentive language, cross-linguistic influence

## Abstract

While understanding and expressing causal relations are universal aspects of human cognition, language users may differ in their capacity to perceive, interpret, and express events. One source of variation in descriptions of caused motion events is agentivity, which refers to the attribution of a result to the agent's action. Depending on the perspective taken, the same event may be described with agentive or non-agentive interpretations. Does language play a role in how people construe and express caused motion events? The present study investigated the use of agentive vs. non-agentive language by speakers of different languages (i.e., monolingual speakers of English and Korean, and Korean learners of English). All three groups described prototypical causal events similarly, using agentive language (active transitive sentences). However, when it came to non-prototypical causal events (where the agent was not shown in the scene), they diverged in their choice of language: English speakers favored agentive language (passive transitive sentences), whereas Korean speakers preferred non-agentive language (intransitive sentences). Korean learners of English patterned with Korean speakers, demonstrating L1 influence on their use of English. These findings highlight the effects of language on motion event construal.

## Introduction

In our everyday life, we talk about causal relations between entities in events, often discussing who did what to whom. While understanding and expressing causal relations are universal aspects of human cognition, individuals differ in their capacity to perceive, interpret, and express events. This is because we do not process everything that comes our way but rather selectively attend to certain input while ignoring the rest. For instance, depending on the perspective we take, the same event of a woman pushing a box can be construed and expressed in different ways, by using an active transitive construction (“The woman pushed the box”), a passive transitive construction (“The box was pushed [by the woman]”), or an intransitive construction (“The box moved”). These three constructions differ in terms of the degree of agentivity and causality, evoking different construals of the same event. In the first two transitive constructions (agentive language), the agent's action is connected to the subsequent result, conflating the action and the result into one macro-event. The agent (*she*) who is held responsible for the result is conceptually salient in both sentence structures although it is less so in the passive construction given its agent-backgrounding nature. In the case of the intransitive construction (non-agentive language), the result enacted is disconnected from the agent's action and is treated as a simplex event (i.e., an event expressed in a single clause that cannot be further divided with resulting subportions). The theme (*the box*) and its change of state receive primary focus, without evoking the causative nature of the action. Given that each of these constructions evokes a different construal of the same event, it can be said that a speaker's choice of construction reflects the perspective (agentive vs. non-agentive) that he/she has taken on the event of interest.

Previous research within functionalist approaches to language has shown that there are various factors that may influence a speaker's choice of sentence structure to describe events in the visual world (e.g., Talmy, 1985; Langacker, 2008). Among many include the attentional salience of a referent (i.e., in terms of the referent's physical salience or the speaker's goals and expectations, e.g., Prentice, 1967), conceptual accessibility (i.e., how easily one can retrieve referential information from working memory, e.g., Bock and Warren, 1985), and prior activation of similar lexical or syntactic units (Bock, 1986). Language is also known to influence the way one expresses and conceptualizes an event in a given discourse. Slobin's (1996); Slobin (2003) thinking-for-speaking hypothesis proposes that language guides our thinking for the purpose of speaking (or other modes of language use) and that speakers are routinely attuned to components of an event that are readily codable in their language. As a result, speakers of typologically different languages tend to express and conceptualize motion events in different ways. These differences can result in some meanings being expressed in some languages while not in others, or being expressed in all languages but with considerable variation in frequency and details provided. For instance, satellite-framed languages (S-languages; e.g., English, German) regularly express information about manner of motion (e.g., walking, crawling) in their motion event descriptions, whereas verb-framed languages (V-languages; e.g., Korean, Spanish) tend to omit this information unless it is particularly salient in the scene (e.g., Talmy, 1985; Slobin, 1996). Additionally, speakers of non-aspect languages (e.g., Dutch, German) prefer to conceptualize motion events in their entirety and encode a possible endpoint of motion, while those of aspect-languages (e.g., English, Spanish) tend to zoom in on the ongoing motion and omit references to event endpoints (e.g., Carroll and von Stutterheim, 2003; Carroll et al., 2004).

Language-specific motion event construal has important implications for additional language learning since second language (L2) learners whose L2 is typologically different from their first language (L1) must learn a new way of selecting and organizing motion components to use their L2 in a target-like manner. Research on cross-linguistic influence in motion event construal has in fact shown that adopting L2-specific construal patterns poses a great challenge to L2 learners. Previous studies focusing on either manner or endpoint encoding patterns (e.g., Carroll and von Stutterheim, 2003; Schmiedtová and Flecken, 2008; Cadierno, 2010; Daller et al., 2011; Larrañaga et al., 2012) have demonstrated that even advanced L2 learners are likely to transfer their L1 construal patterns to their L2 use, a linguistic phenomenon widely known as conceptual transfer (or (re)thinking-for-speaking; see Jarvis, 2016 for a discussion of how these two frameworks differ in their scope of research). Although there is some evidence for L2 learners' partial convergence to the target-like conceptualization patterns (e.g., von Stutterheim, 2003; Cadierno, 2004; Cadierno and Ruiz, 2006; Park, 2020a), research to date generally substantiates that learners' routinized and entrenched L1 construal patterns are likely to exert an influence on their L2 use.

While there has been a growing interest in examining motion event construal in L2 or bilingual speakers, the scope of this research has been mostly confined to the domain of spontaneous motion events, particularly in relation to the encoding of manner or endpoint. Relatively less attention has been devoted to exploring conceptual transfer emerging from any other cross-linguistic differences besides those related to manner or endpoint encoding. In fact, recent research on event construal has shown that language-specific narrative habits are evident in the way people express causation (e.g., Gullberg, 2009; Fausey and Boroditsky, 2011; Luk, 2014; Cadierno et al., 2016; Okuno et al., 2020; Lewandowski and Özçalişkan, 2021). The domain of caused motion is an ideal testing ground for exploring motion event construal as it presents a number of interesting cross-linguistic properties. For instance, some studies have shown that languages such as English prefer agentive interpretations of caused motion events and tend to give prominence to agents while others (e.g., Japanese, Korean, Spanish) orient toward non-agentive interpretations and are likely to background agents (Choi, 2009; Fausey and Boroditsky, 2011; Filipović, 2013; Luk, 2014; Okuno et al., 2020). This type of typological contrast between languages may offer new insights into ongoing research on conceptual transfer, extending the scope of research beyond the encoding of manner and endpoint reference.

To advance the emerging line of research on the role of language in agentivity, the present study partially replicated and extended upon Choi (2009), which among others examined the way in which first language (L1) speakers highlighted agentivity and causality in their motion event descriptions. By extending her work to the second language (L2) context, the present study sought to compare how L2 speakers with two typologically different languages construed and described motion event scenes in comparison to their monolingual counterparts. English and Korean were chosen as a language pair because these two languages are known to place different constraints on the way they encode information about causal relations, namely, with respect to assigning agency and highlighting causation (Kim, 2006; Choi, 2009; Wolff et al., 2009). Of particular interest was to examine the extent to which native speakers of Korean, native speakers of English, and Korean learners of English would make use of agentive vs. non-agentive language in their descriptions of motion events under investigation.

## Previous Research

### Different Expressions, Different Construals

**Figure 1:**
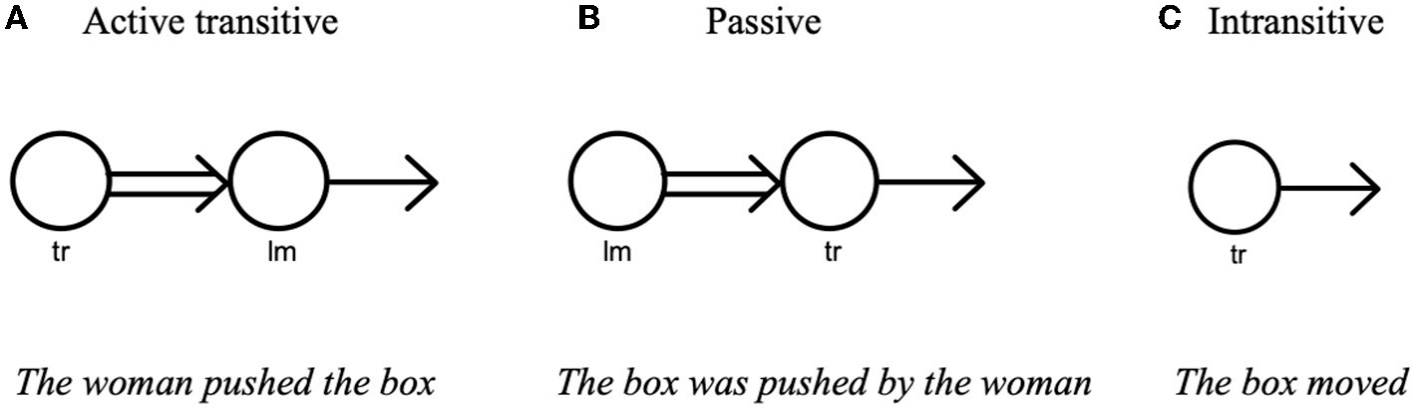
Profiling of different constructions (Langacker, 2008, p. 385)—From the left, it should be labeled **(A–C)**. Bold lines indicate the aspect of the event being profiled; the double arrows represent the exertion of force; the single arrows represent a change of state; “tr” and “lm” stand for “trajector” and “landmark,” respectively.

In Cognitive Grammar, the meaning of an expression is closely tied to how the speaker construes an event (Langacker, 2008). While construal is grounded in general cognitive abilities, the speaker's linguistic choices (e.g., verb use, syntactic structure) largely depend on how much information is available to the speaker and what one wishes to reveal about the event. Thus, one's conceptualization of the event is reflected in the way in which event components are linguistically encoded. For instance, a direct causative event^1^ such as *the woman pushing the box* can be construed and expressed in multiple ways within a single language. If we conceptualize the relations that exist between the two entities (i.e., *the woman* and *the box*) as causal, we are likely to use an active transitive construction (i.e., the agent-action-theme^2^ schema) to describe this event as in (1a). A causal relationship is understood as an event in which one entity (the causer or agent—*the woman*, in this case) exerts an external force to bring about a change of state in another entity (the theme—*the box*) (Talmy, 2000). A prototypical agent is an animate, sentient being with consciousness and volition that carries out an action (Taylor, 1995; Langacker, 2008) and figures as the subject. In Langacker's (2008) term, the agent that receives primary focus is called trajector, and the theme with secondary focus is called landmark. As illustrated by the image schema in Figure 1A, an active transitive sentence evokes and profiles (noted with heavy lines in the figure) both an agent's exertion of force as well as the thematic process it brings about.

When defocusing of an agent is needed (e.g., the agent is visually less prominent in the scene, and thus, his/her identity is unknown, irrelevant, or best concealed), it is plausible to use a passive (transitive) construction, as in (1b). While the passive evokes the entire agent-theme interaction^3^, it differs from the active transitive sentence in that it selects the theme as trajector by backgrounding the agent in the conceptual structure of the event (Langacker, 2008). When the conceptual backgrounding of the agent is strong, the *by*-phrase with agentive information (*by the woman*) is left unmentioned in the passive construction^4^.

Lastly, the speaker may choose to conceptualize *the woman pushing the box* as spontaneous motion rather than as caused motion in certain contexts (e.g., the agentive process is not clearly shown in the scene) and describe the event with a non-agentive intransitive construction as in (1c). In this case, neither the agent nor the agent's exertion of force is evoked in the conceptual realm; the thematic process is conceptualized autonomously without reference to an agent or agentive causation. Thus, the non-agentive theme receives focal prominence as trajector. As shown in Figure 1C, the theme's change of state is made explicit as the processual profile without any specified landmark. The intransitive construction contrasts with the passive construction, in which the agent is not mentioned but nevertheless still implied.

(1a) The woman pushed the box.
(1b) The box was pushed (by the woman).
(1c) The box moved.

In sum, language is our basic means for describing the outside world, and different linguistic descriptions reflect different construals of the same event. Central to perceiving and encoding events are the relations between the entities involved in them. The way in which we identify agents, themes, and their relation in events will guide our selection of linguistic descriptions. While active and passive sentences differ in the degree of prominence conferred on participants involved in the event, they are similar in that each evokes the agent and agentive causation. Thus, it is appropriate to refer to them as agentive language. Unlike these two constructions, the intransitive construction takes a non-agentive perspective by framing the event in question as a non-causal, spontaneous event. In this respect, it can be said that the three constructions (i.e., the transitive, passive, and intransitive constructions) are situated at three different points on a continuum, which reflects a gradual decrease in the degree of agentivity and causality from left to right (Ikegami, 1991).

### Cross-Linguistic Differences in the Preference for Different Constructions

To date, there is only a handful of studies that have shown that some languages prefer agentive interpretations of caused motion events while others orient toward non-agentive interpretations. For instance, Fausey and Boroditsky (2011) and Filipović (2013, 2018, 2022) demonstrated that when an agent caused an action with a clear intention, both English and Spanish speakers used agentive descriptions to give prominence to intentional actions (e.g., English = *The girl popped the balloon*, Spanish = *La muchacha rompió el globo*). However, when the agent's action was clearly accidental, Spanish speakers preferred to provide explicit information about the action being accidental (e.g., *Se le rompió el globo a la muchacha* “To-her-the-balloon-burst to the girl”) while English speakers did not specify this information in their descriptions (e.g., *The girl popped the balloon*). This suggests that the former made significantly more non-agentive interpretations than the latter. Similarly, other studies (Luk, 2014; Okuno et al., 2020) have reported that English and Japanese differ in how much prominence they provide to an agent: English tends to give prominence to a human agent and make frequent use of transitive sentences while Japanese prefers to suppress the human agent and make wide use of intransitive sentences.

Consistent with these findings is the investigation made by Choi (2009), which investigated similarities and differences in the way typologically different languages (i.e., English, Japanese, Korean, Spanish) encode path and cause of motion. Among others, the study explored the extent to which causality becomes highlighted in S-language and V-language speakers' linguistic descriptions. This investigation was done rather in an exploratory manner since only four of the 28 stimuli employed were dedicated to examining speakers' sensitivity to agentivity. It was hypothesized in Choi (2009) that S-language (English) speakers would highlight causal aspects more than V-language (Japanese, Korean, Spanish) speakers because S-languages encode Cause in the main verb while V-languages encode Path. To test this hypothesis, Choi (2009) provided participants with scenes that would give them a choice about what to highlight in their linguistic expression. When event scenes depicted the human agent causing the entity's movement (*k* = 2), participants regardless of their language backgrounds highlighted causation in their motion event descriptions by employing transitive sentences. However, when event scenes only showed the entity's movement without the agent causing it (*k* = 2), the choice of construction diverged between S-language and V-language speakers. English speakers primarily used agentive descriptions (both transitive and passive sentences), while V-language speakers made more use of non-agentive descriptions (intransitive sentences). Korean speakers, in particular, contrasted the most with English speakers, displaying a strong preference for non-agentive language. Although the observed cross-linguistic differences were based on the small number of stimuli (two scenes per condition), the study nevertheless revealed interesting insights into how typologically different languages highlight agentivity and causality to varying degrees.

The agentive/non-agentive divergence between English and Korean can be further explained by Ikegami's (1991) language typology. According to Ikegami (1991), languages differ by which they prefer along the “transitive-passive-intransitive” continuum. There are languages such as English (called DO-languages) that prefer to focus on an individual, especially a human being, and give linguistic prominence to the notion of agency. These languages make high use of the transitive construction because (a) it offers potentially two argument positions (i.e., the subject and object positions) in which a human entity may be linguistically coded (c.f., the intransitive frame requires only one argument), and (b) the “transitive” agent is higher in the degree of agentivity than the “intransitive” agent as the effects of the agent's action affects another entity in the former. Even in cases in which an agent needs to be suppressed, DO-languages are likely to choose the passive construction over the intransitive construction to indicate the presence of an implied agent at a minimum (Ikegami, 1991). Contrarily, languages such as Japanese and Korean (called BECOME-languages) have a tendency to suppress the notion of agency and focus on the event as a whole. Thus, BECOME-language users make use of a variety types of agent backgrounding constructions such as agentless transitive sentences (this is acceptable only in pro-drop languages), passive sentences, and intransitive sentences, for their linguistic expressions.

Empirical evidence showing that Korean tends to make more use of non-agentive language than English comes from several cross-linguistic studies of corpus data. For instance, Kim (2015) compared English articles from the *Reader's Digest* magazine and Korean translational equivalents and demonstrated that English passive sentences were frequently translated into Korean as intransitive sentences as in (2).

(2a) John was killed in the accident.
(2b) 존은 그 사고로 죽었다.
con-un ku sako-lo cwuk-ess-ta.
John-TOP that accident-due to die-PAST-DC^5^
“John died in the accident”

Similarly, Kim (2006) compared transitive sentences extracted from English *CNN* newspapers with their Korean translational equivalents and reported that English transitive sentences with inanimate subjects were translated into Korean primarily as passive and intransitive sentences. The use of intransitive sentences in this case can be seen as an avoidance strategy because Korean does not easily permit inanimate entities to feature as the subject of a transitive construction (Kim, 2006; Wolff et al., 2009). In fact, acceptable subjects in Korean are typically animate entities. English, however, allows an extension of the notion of agency to entities with no agentive properties as shown in (3), permitting a wide variety of inanimate entities to feature as the agent of causal expressions. Ikegami (1991) remarks that “English has far less difficulty with this rhetorical operation because the ‘actor-action' construction is its favorite (p. 313).” As a result, transitive sentences are more productive in English than in Korean, and the opposite is true for non-agentive constructions.

(3a) The lightning destroyed the building.
(3b) Liquor killed him.

While there has been relatively little attention placed on motion event construal concerning agentivity, scant research to date suggests that languages may vary in their preference for agentive vs. non-agentive language. However, it should be noted that empirical evidence attesting to the typological difference between English and Korean primarily comes from corpus data containing English sentences extracted from written sources and their Korean translational equivalents. Thus, more research examining language users' actual descriptions of caused motion events, such as Choi (2009), seems warranted. Furthermore, most research on agentive vs. non-agentive language, including Choi (2009), has been limited to the study of monolingual speakers, and as a result, little is known about the extent to which L2 speakers highlight agentivity in comparison to their monolingual counterparts. Thus, it seems necessary to extend the scope of work on agentivity to the L2 context, which in turn will provide new insights into ongoing research on conceptual transfer.

## Research Questions

To supplement the current dearth of cross-linguistic studies dealing with agentive vs. non-agentive language, the present study sought to investigate how speakers of different languages, specifically Korean speakers, English speakers, and Korean English-as-a-foreign-language (EFL) learners, conceptualize and describe event scenes that varied in the degree of agentivity and causality. The present study partially replicated and expanded on Choi (2009) to further explore how the presence (or lack thereof) of an (animate) agent responsible for an entity's movement affected participants' choice of construction. It is a replication in the sense that it adapted Choi's (2009) experimental conditions with more stimuli. The study contrasted events where an agent causing an entity's movement was visible (the Agent Visible condition) against events where the agent was not visible (the Agent Invisible condition). It further extended upon her work by employing a finer-grained analysis of motion event descriptions (see Section Coding for more information) and exploring the preferred choice of construction for Korean EFL learners in their L2 motion event descriptions. The present study was guided by the following research questions (RQs):

1. To what extent was agentivity highlighted in participants' descriptions of Agent Visible videos?
2. To what extent was agentivity highlighted in participants' descriptions of Agent Invisible videos?

Based on the findings of Choi (2009), it was hypothesized that both Korean and English speakers would employ transitive constructions to describe Agent Visible videos. Since no cross-linguistic differences were to be expected between the two monolingual groups, it was hypothesized that Korean EFL learners would also highlight causation in their descriptions to a comparable degree by employing transitive sentences. In contrast, more variations were to be observed in the choice of sentence type in the Agent Invisible condition because there were several options (e.g., the passive sentence, the transitive sentence) at hand to background an agent. It was predicted that Korean speakers would utilize intransitive sentences more frequently than English speakers due to their tendency to suppress an agent in their descriptions. On the basis of previous studies on conceptual transfer (e.g., Cadierno, 2010; Larrañaga et al., 2012; Park et al., 2022), it was further hypothesized that EFL learners would display L1-entrenched preferences in their L2 descriptions.

## Method

### Participants

A total of 101 participants were recruited for the present study: 14 Korean monolinguals (KM; age, *M* = 28, *SD* = 7.40), 14 English monolinguals (EM; age, *M* = 21.93, *SD* = 4.89), and 73 Korean EFL learners (age, *M* = 22.67, *SD* = 2.78). All Korean-speaking participants were recruited from universities and local churches in Korea, and English participants were recruited from universities in the U.S. Although Korean speakers reported having learned English as part of their formal education, they identified themselves as Korean monolingual speakers at the time of testing. Statistical analyses indeed confirmed that Korean speakers' self-reported proficiency level in English (*M* = 6.60 out of 20, *SD* = 3.27) and their English test (Elicited Imitation Task; Wu et al., 2022) scores (*M* = 28.60 out of 120, *SD* = 10.94) were significantly lower than those of Korean EFL learners. Most EFL learners were sequential learners whose learning of English began after the age of five (only seven of them reported an early age of onset, before the age of five). They represented a wide range of English proficiency levels according to their self-reported proficiency level in English (*M* = 13.23 out of 20, *SD* = 2.72) and English Elicited Imitation Task scores (*M* = 77.96 out of 120, *SD* = 15.19).

### Materials

#### Video Description Task

Following Choi (2009), a video description task (*k* = 10) was developed using four causative verbs (*drop, pour, roll, throw*) to elicit participants' verbal descriptions of motion events. Two of those verbs (roll, throw) were from Choi (2009), and two other verbs that elicited similar caused motion (an agent exerts a force on an inanimate object, which in turn, moves to a different location) were selected. Five target events were filmed in two different versions: In one version (the Agent Visible condition), participants saw a woman directly causing an inanimate object to move (e.g., *the woman rolls the ball into the bag*), and in another (the Agent invisible condition), they saw the same object's movement without the agent responsible for the action. In other words, it appeared as though the object acted on its own. Table 1 provides the list of descriptions for these 10 scenes. Because this study was part of a larger project, there were 44 other stimuli scenes that served as fillers for the 10 critical items. The number of fillers occurred between critical items ranged from 1 to 10, and the two versions of the same target scenes were separated by more than 10 fillers (min = 14 fillers, max = 56 fillers). The total of 54 stimuli items were presented in a fixed random order to prevent items of the same type from appearing in adjacent positions. Each video clip lasted about 7 s, and participants were instructed in their native language to describe what was happening in each video (English = “Describe what is happening in the video”, Korean = “비디오 속에서 일어나는 일을 설명하시오”). While participants were allowed to replay a video clip as many times as they wished, backtracking to a previously responded item was not permitted. The EFL learners were provided with a list of key nouns and the Korean definitions, which they could consult while completing the task.

**Table 1:** Event stimuli.

**Action**	**Agent visible condition**	**Agent invisible condition**
Throw pen	Woman throws pen into container	(Woman) throws pen into container
Roll ball	Woman rolls ball into plastic bag	(Woman) rolls ball into plastic bag
Pour rice	Woman pours rice into container	(Woman) pours rice into container
Drop cellphone	Woman drops cellphone into bag	(Woman) drops cellphone into bag
Drop pen	Woman drops pen into basket	(Woman) drops pen into basket

#### Background Questionnaire

An online background questionnaire was developed using Qualtrics (www.qualtrics.com) and administered to all participants to gather information about their language learning experience including self-reported estimations of English proficiency and knowledge of other foreign languages.

#### English Proficiency Measurement: Elicited Imitation Task

Korean participants' English proficiency was measured with an elicited imitation task (Wu et al., 2022) that estimated participants' ability to repeat oral sentences. The task consisted of 30 English sentences, and participants had to listen to one sentence at a time and repeat the sentence as accurately as possible. The participants' responses were recorded and then coded using a five-point scoring rubric (Ortega et al., 2002). The maximum score was 120.

### Procedure

The study took place in a single session in a laboratory after the participants had completed the online background questionnaire at home. All participants met with the researcher individually and completed the video description task. The elicited imitation task was administered only to the Korean participants.

### Coding

While Choi (2009) coded the data largely into two categories, causative/agentive (transitive and passive constructions) and non-causative/non-agentive (intransitive constructions), the present study employed a finer-grained coding scheme to capture a fuller degree of agentivity. Each video description was coded as “active,” “passive,” or “intransitive.” Active descriptions included an agent as the subject and a theme as in, “The woman threw the pen into the box.” Korean transitive sentences were further coded into two categories based on the presence of an overt agent ([+subject] and [-subject]). For instance, “여자가 펜을 던졌다 (The woman threw the pen)” was coded as [+subject], while “펜을 던졌다 (threw the pen)” was categorized as [-subject]. Sentences that featured the theme as the subject with the transitive verb in past participle form were coded as passive descriptions. Descriptions were coded as intransitive if the theme served as the subject followed by an intransitive verb. Descriptions that did not fit into one of the three types were coded as “other” sentences. Table 2 includes example descriptions for each sentence type extracted from English and Korean dataset. To establish reliability, a trained Korean-English bilingual assistant double-coded 15% of the descriptions. A high level of agreement was obtained for both the English and Korean data (for English, Cronbach's alpha = 0.95; for Korean, Cronbach's alpha = 0.96).

**Table 2:** Example descriptions extracted from English and Korean dataset.

**Type**	**Agentivity**	**English**	**Korean**
Transitive	Strong	The woman threw the pen into the box.	여자가 펜을 던져 넣었다. yeca-ka pheyn-ul tency-e neh-essta. Woman-NOM pen-ACC throw-and put-PAST-DC “The woman put the pen, throwing”
	Less strong	N/A	그릇 안에 펜을 던졌다. Kulus an-ey pheyn-ul tency-ess-ta. Container inside-LOC pen-ACC throw-PAST-DC “(The woman) threw the pen inside the container”
Passive^a^	Weak	A pen is thrown into a plastic container.	형광펜이 가방 안으로 던져지다. Hyengkwangpheyn-i kapang an-ulo tency-e-ci-ta Highlighter-NOM bag inside-LOC throw-PASS-DC “The highlighter was thrown into the bag”
Intransitive	Zero	A pen falls into a purse.	가방 안으로 펜이 떨어진다. Kapang anulo pheyni ttelecinta. Bag inside-LOC pen-NOM drop-PRES-DC “The pen drops into the bag”
Other	Zero	The phone is in the bag.	“Other” sentences were not found in the Korean dataset.

*Abbreviations for glosses used in the examples are: ACC, accusative case; DC, declarative sentence-type suffix; LOC, locative case; NOM, nominative case; PASS, passive; PAST, past tense; PRES, present tense*.

*^a^The coding scheme does not include passive sentences with a by-phrase because no such sentence was found in my dataset*.

## Results

**Figure 2:**
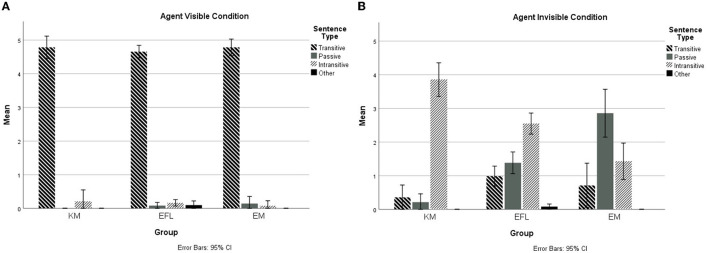
Frequency of different sentence types in Agent Visible **(A)** and Agent Invisible **(B)** conditions.

Group means and standard deviations for the overall number of responses coded as active, passive, intransitive, and other sentences in the Agent Visible and Agent Invisible conditions are provided in Table 3 and graphically represented in Figure 2.

**Table 3:** Descriptive statistics: group means (and standard deviations).

**Agent Visible Condition**				
		**KM (*****n*** **=** **14)**	**EFL (*****n*** **=** **73)**	**EM (** ***n** **=*** **14)**
Transitive	[+subject]	2.21 (2.23)	4.66 (0.80)	4.79 (0.43)
	[-subject]	2.57 (2.21)	N/A	N/A
	Total	4.79 (0.58)	4.66 (0.80)	4.79 (0.43)
Passive		0	0.08 (0.40)	0.14 (0.36)
Intransitive		0.21 (0.58)	0.16 (0.41)	0.07 (0.27)
Other		0	0.10 (0.53)	0
**Agent Invisible Condition**				
	KM	EFL	EM
Transitive	[+subject]	0.36 (0.63)	0.99 (1.28)	0.79 (1.12)
	[-subject]	0.57 (0.76)	N/A	N/A
	Total	0.93 (0.73)	0.99 (1.28)	0.79 (1.12)
Passive		0.21 (0.43)	1.38 (1.38)	2.71 (1.44)
Intransitive		3.86 (0.86)	2.55 (1.34)	1.50 (1.09)
Other		0.08 (0.32)	0	0

*KM, the Korean monolingual group; EM, the English monolingual group; EFL, the EFL group; N/A, Not applicable*.

As expected, participants across groups almost always produced transitive sentences in the Agent Visible condition. The results of a one-way ANOVA with Group as the sole independent variable confirmed that there was no statistically significant group difference, *F*_(1, 99)_ = 0.54, *p* = 0.47. In using transitive sentences, Korean speakers employed [+subject] and [–subject] descriptions to a comparable degree according to paired *t*-test results, *t*_(13)_ = −0.30, *p* = 0.77. Participants rarely produced passive, intransitive, or any other sentence forms to describe caused events with a visible agent. The only group that described Agent Visible scenes with “other” sentences was the EFL group (just four cases), and these sentences all referred to the final state of the inanimate object (e.g., *There is a ball inside the basket*).

More variability was observed in how participants described scenes in the Agent Invisible condition. The most preferred sentence type for the KM and EFL groups was the intransitive sentence while EM group favored passive sentences. None of the groups preferred the transitive sentence, which was their primary choice of sentence type for Agent Visible scenes. A one-way MANOVA was performed, with three dependent variables (transitive, passive, and intransitive sentences) and one independent variable (Group). Other sentences were not included in the analysis as they were minimally employed by the EFL group only. Using Pillai's Trace, the results showed a statistically significant difference across groups on the combined dependent variables, *F*_(6, 194)_ = 5.00, *p* < 0.001, *V* = 0.27, partial η^2^ = 0.13. Separate univariate ANOVAs revealed that Group had a statistically significant effect on passive sentences, *F*_(2, 21.94)_ = 12.92, *p* < 0.001, partial η^2^ = 0.21, and intransitive sentences, *F*_(2, 19.59)_ = 12.21, *p* < 0.001, partial η^2^ = 0.20. Therefore, *post-hoc* analyses were conducted using a Bonferroni adjusted alpha level of 0.017. For passive sentences, statistically significant differences were found among all three comparisons: KM-EM (*p* < 0.001, *d* = −2.36), KM-EFL (*p* = 0.008, *d* = −1.15), and EM-EFL (*p* = 0.002, *d* = 0.94). Similarly, three groups also differed from each other in their use of intransitive sentences: KM-EM (*p* < 0.001, *d* = 2.40), KM-EFL (*p* = 0.002, *d* = 1.14), and EM-EFL (*p* = 0.015, *d* = −0.86).

To investigate whether the EFL learners' use of passive and intransitive constructions (i.e., the two construction types in which the KM and EM groups diverged the most) was mediated by their proficiency level, a Pearson correlation was conducted on the frequency of passive and intransitive sentences and EFL learners' self-rated proficiency scores and EIT scores. The results, however, indicated that no statistically significant relationship was found between the EFL learners' use of passive and intransitive sentences and their proficiency scores.

## Discussion

The first research question examined the extent to which participants highlighted agentivity in their descriptions of motion event scenes that featured an agent causing an entity's movement. It will be remembered that these scenes clearly depicted the agent's action and the affected theme's movement, allowing viewers to readily assume the clausal relationship between the two entities (the animate agent and the inanimate theme) and to express the scenes with agentive descriptions. Consistent with the findings of Choi (2009), all three language groups predominantly employed transitive descriptions to a comparable degree. These results suggest that people are likely to highlight agentivity and causation in their descriptions when viewing prototypical caused motion events. While the agent features as the subject in the transitive sentence in both English and Korean, it must be remembered that there is a way to demote an agent even in transitive sentences in Korean. Being a pro-drop language and discourse-prominent language (Clancy, 1996; Kim, 1997), Korean allows referents to be omitted when they are sufficiently recoverable from the immediate discourse context. For instance, if the referent has been already introduced in the previous sentence and is thus active at the time of utterance, the subject is likely to be omitted. Interestingly, the video description task in the present study did not present scenes as a connected event (critical items were, in fact, intermingled with distractor items that featured a different female person), meaning there was no immediate discourse context to refer back to. Nevertheless, the Korean speakers made use of agentless transitive sentences approximately half of the time. These findings demonstrate that Korean speakers' tendency to suppress an agent was also at work despite the fact that the agent of caused motion was visually prominent in the scene.

The second research question explored the extent to which participants highlighted agentivity in their descriptions of motion event scenes that did not feature an agent causing an entity's movement. Based on the results of Choi (2009), it was expected that more variations would be observed in the choice of sentence type in the Agent Invisible condition because there were several options (e.g., the passive sentence, the intransitive sentence) at hand to background an agent. As predicted, the Korean speakers suppressed an agent to a greater degree compared to the English speakers: While the Korean speakers predominantly favored intransitive sentences, the English speakers showed a preference toward passive sentences. As illustrated in Figure 1, the main difference between the passive and intransitive construction is that the former conceptualizes an event as a caused motion event and evokes an implied agent while the latter conceptualizes an event as a spontaneous motion event and does not evoke an agent in the conceptual realm. As a result, both sentence structures are treated as agentive language. In the case of intransitive sentences, the non-agentive theme receives focal prominence without reference to an agent or agentive causation. The contrasting tendencies observed between the two monolingual groups corroborate Ikegami's (1991) claim that DO-languages and BECOME-languages differ in the extent to which they highlight agentivity in their linguistic descriptions. As DO-language users, English speakers tend to give prominence to agentivity while Korean speakers, as BECOME-language users, tend to de-emphasize agentivity. This finding substantiates Slobin (1996, 2003) thinking-for-speaking hypothesis, which posits that language-specific patterns direct the speaker's attention to specific aspects of motion events for the purpose of speaking.

When the EFL learners were instructed to describe Agent Invisible videos in L2 English, their most preferred choice of construction aligned with that of the Korean speakers, that is, intransitive sentences. This suggests that the EFL learners transferred their L1 patterns in their use of the L2, displaying conceptual transfer. Such results are not particularly surprising given that their dominant language at the time of testing was Korean. According to Montrul (2016), language dominance refers to “the relative weight and relationship of the two languages in terms of language use and degree of proficiency” (p. 16). Although no direct measure of language dominance was employed in the present study, it seems reasonable to assume that the EFL learners' dominant language was Korean based on the fact that they were mostly sequential L2 learners who grew up in Korea, started learning English after the age of 5, and were fully proficient in Korean while their English proficiency level varied greatly. Thus, it is possible that the effects of language dominance, together with the effects of language of instruction (i.e., language in which the instructions were given, which was Korean for the EFL learners), might have triggered a stronger L1 influence on their choice of constructions. Future research should consider further examining the role of language dominance in L2 learners' choice of construction by replicating the present findings with Korean-English speakers whose dominant language is English.

Previous research has shown that a variety of factors may shift L2 learners' behavior toward either L1 or L2 patterns, including language mode (e.g., monolingual vs. bilingual mode, Berthele and Stocker, 2017), L2 proficiency (e.g., Treffers-Daller and Calude, 2015; Park, 2020a,b), and language dominance (e.g., Daller et al., 2011). However, the present study indicated that the EFL learners' use of agentive vs. non-agentive language did not vary depending on their English proficiency determined by EIT scores and self-rated proficiency. This suggests that there may be other factors besides L2 proficiency, which the present study has admittedly failed to capture, that could have accounted for the variation observed in the EFL learners' choice of construction. It may be worthwhile to examine how cultural factors such as the degree of exposure to L2 culture or the relative value placed on interdependence and independence influences L2 speakers' choice of sentence structure and perspective taking. The tendency to efface or highlight the notion of agentivity in speakers' language use may originate from their deeply ingrained cultural attitudes and beliefs. For instance, East Asians cultures tend to endorse more interdependent cultural norms, placing a high value on group harmony, relatedness, and connections with others, whereas Western culture shows the opposite patterns as they endorse more individualistic cultural norms that highlight self-direction, autonomy, and self-expression (Varnum et al., 2010). Previous studies (e.g., Masuda and Nisbett, 2001; Nisbett, 2010) have shown that these differences in cultural norms may, in fact, affect the way in which speakers construe and express motion events. East Asians tend to be holistic, focusing on a salient focal object and the context to which it belongs. In contrast, Westerners tend to be analytic, attending to the object in relative isolation from the context in which it is embedded. Thus, it may be possible that L2 speakers' preference for a certain construction type may be mediated by the extent to which they are exposed by Western culture that values independence or their cultural attitudes toward interdependence and independence. Future research that employs a direct measure of exposure to L2 culture or cultural attitudes is warranted to further investigate factors that may influence L2 speakers' preference for sentence structures. Additionally, future studies should consider employing designs with individuals that have similar cultural norms but whose L1s present interesting cross-linguistic differences in relation to preferred patterns of expression, to tease apart linguistic and cultural effects on event construal.

## Conclusions

Depending on the perspective taken, the same event may be construed and described in different ways. While construal is grounded in general cognitive abilities, the speaker's linguistic choices may vary depending on what one wishes to reveal about the event. The present study partially replicated and extended upon Choi (2009) with the goal to investigate how language may influence the way in which people construe and describe caused motion events. By demonstrating that Korean speakers preferred to employ sentences that give less prominence to agentivity and causality than English speakers in both experimental conditions, the present study confirms that sentence structure preferences may vary as a function of language (Ikegami, 1991). These findings are well-aligned with a large body of empirical evidence in support of the thinking-for-speaking hypothesis accumulated over the last two decades, which suggests that speakers of different languages select and attend to different types of information as revealed by their verbal expression as well as non-verbal behavior. Korean EFL learners, whose dominant language was Korean, patterned with their L1 monolingual counterparts in their choice of sentence structure, displaying conceptual transfer in their L2 descriptions of motion events. This finding lends support to the claim that routinized L1 patterns are highly resistant to restructuring (Slobin, 1996; Langacker, 2008).

## Data Availability Statement

The raw data supporting the conclusions of this article will be made available by the authors, without undue reservation.

## Ethics Statement

The studies involving human participants were reviewed and approved by Georgetown University. The patients/participants provided their written informed consent to participate in this study.

## Author Contributions

The author confirms being the sole contributor of this work and has approved it for publication.

## Conflict of Interest

The author declares that the research was conducted in the absence of any commercial or financial relationships that could be construed as a potential conflict of interest.

## Publisher's Note

All claims expressed in this article are solely those of the authors and do not necessarily represent those of their affiliated organizations, or those of the publisher, the editors and the reviewers. Any product that may be evaluated in this article, or claim that may be made by its manufacturer, is not guaranteed or endorsed by the publisher.

## Footnotes

^1^Direct causation refers to an event in which a causer directly effects a causee by means of physical contact. While direct causation can be expressed with both a lexical causative and a periphrastic causative, the current study only focuses on direct causation expressed with a lexical causative in a single clause and includes a causer, causee, and a change of state (e.g., *The woman pushed the box*). Thus, the discussion of periphrastic causatives that are composed of two clauses (e.g., *The woman caused the child to push the box)* is beyond the scope of this study.

^2^In Cognitive Grammar, the term *theme* subsumes various passive (i.e., it is not construed as a source of energy) semantic roles including *patient, mover, experiencer*, and *zero* (Langacker, 2008). Thus, the current study uses *theme* for what is typically referred to as *patient*.

^3^This is why there always exists the feeling that the action has been carried out by somebody even if the agent is not overtly expressed with the *by-*phrase.

^4^If the by-phrase in the passive construction is omitted, the image schema in Figure 1B needs to be modified as landmark the woman is no longer being profiled. In that case, the left circle representing the woman needs to be unbolded.

^5^Abbreviations for glosses used in this example are: DC, declarative sentence-type suffix; PAST, past tense; TOP, topic marker.
